# Stimulus Design for Model Selection and Validation in Cell Signaling 

**DOI:** 10.1371/journal.pcbi.0040030

**Published:** 2008-02-15

**Authors:** Joshua F Apgar, Jared E Toettcher, Drew Endy, Forest M White, Bruce Tidor

**Affiliations:** 1 Department of Biological Engineering, Massachusetts Institute of Technology, Cambridge, Massachusetts, United States of America; 2 Computer Science and Artificial Intelligence Laboratory, Massachusetts Institute of Technology, Cambridge, Massachusetts, United States of America; 3 Center for Cancer Research, Massachusetts Institute of Technology, Cambridge, Massachusetts, United States of America; 4 Department of Electrical Engineering and Computer Science, Massachusetts Institute of Technology, Cambridge, Massachusetts, United States of America; Lawrence Berkeley National Laboratory, United States of America

## Abstract

Mechanism-based chemical kinetic models are increasingly being used to describe biological signaling. Such models serve to encapsulate current understanding of pathways and to enable insight into complex biological processes. One challenge in model development is that, with limited experimental data, multiple models can be consistent with known mechanisms and existing data. Here, we address the problem of model ambiguity by providing a method for designing dynamic stimuli that, in stimulus–response experiments, distinguish among parameterized models with different topologies, i.e., reaction mechanisms, in which only some of the species can be measured. We develop the approach by presenting two formulations of a model-based controller that is used to design the dynamic stimulus. In both formulations, an input signal is designed for each candidate model and parameterization so as to drive the model outputs through a target trajectory. The quality of a model is then assessed by the ability of the corresponding controller, informed by that model, to drive the experimental system. We evaluated our method on models of antibody–ligand binding, mitogen-activated protein kinase (MAPK) phosphorylation and de-phosphorylation, and larger models of the epidermal growth factor receptor (EGFR) pathway. For each of these systems, the controller informed by the correct model is the most successful at designing a stimulus to produce the desired behavior. Using these stimuli we were able to distinguish between models with subtle mechanistic differences or where input and outputs were multiple reactions removed from the model differences. An advantage of this method of model discrimination is that it does not require novel reagents, or altered measurement techniques; the only change to the experiment is the time course of stimulation. Taken together, these results provide a strong basis for using designed input stimuli as a tool for the development of cell signaling models.

## Author Summary

A major focus of systems biology is the development of mechanism-based models of cell signaling pathways. These models hold the promise of encapsulating our understanding of complex biological processes while also predicting new behavior. However, as these models become more complex, it can be difficult to distinguish between model alternatives. One means of improved model discrimination involves making measurements of additional components in the biological system to provide more detailed data. Here we present an alternative, which is to apply a time-varying input while monitoring the same network components. This new method was able to discriminate among models with subtle mechanistic differences. A particular advantage is that for many cases, time-varying input stimulation is fairly easy to apply experimentally, whereas measuring additional network components can involve the creation of new reagents or measurement assays. Thus, we believe that the application of time-varying input stimulation will become a powerful tool in the field of systems biology as the community places increased emphasis on the development of quantitative, mechanistic, and predictive models of biological network behavior.

## Introduction

One goal of systems biology is to develop detailed models of complex biological systems that quantitatively capture known mechanisms and behaviors, and also make useful predictions. Such models serve as a basis for understanding, for the design of experiments, and for the development of clinical intervention. In support of this goal, there has been a strong push to build mechanistically correct kinetic models, often based on systems of ordinary differential equations (ODEs), that are capable of recapitulating the dynamic behavior of a signaling network. These models hold the promise of connecting biological and medical research to a class of computational analysis and design tools that could revolutionize how we understand biological processes and develop clinical therapies [1,2].

One type of experiment for model validation involves stimulating a system with a step change in the input (typically by adding a high concentration of ligand) and then measuring the change of network readouts (the concentrations or activities of various downstream species) as a function of time. Candidate models are fit to the data and the best model is selected based on criteria such as the quality of the fit, the simplicity of the model, and other factors. While it is tempting to select a simple model consistent with the known biochemical mechanisms that fits all available data, future experimentation may prove this choice incorrect. Rather, it may be preferable to collect “all” models consistent with known mechanisms and data, and to design follow-on experiments capable of distinguishing among the model candidates. In support of this less-biased approach, here we develop an approach for designing these follow-on experiments using dynamic stimuli.

While the step-response experiment is attractive for its ease of implementation, dynamic stimuli have the potential to uncover more subtle system dynamics and to improve model selection in the cases where step-response experiments are not sufficiently discriminating. One example that illustrates the use of a dynamic stimulus to distinguish between two models is the work by Smith-Gill and co-workers on the detailed mechanism of antibody–antigen binding [3]. Initial step-response experiments were compatible with either a one-step or two-step binding mechanism, in which the ligand and antibody first come together in a loose encounter complex before forming a fully bound complex. To resolve this ambiguity, the authors applied a series of rectangular pulses of ligand concentration to their system. The resulting binding curves produced by this dynamic stimulus were inconsistent with the one-step model but were consistent with a two-step model and suggested the existence of an encounter complex, even though such a complex could not be measured directly by the assay.

These results show that time varying inputs have the potential to distinguish closely related models of biochemical systems. For the relatively simple antibody–antigen system, an appropriate dynamic input was deduced intuitively. However this sort of intuitive design is difficult, especially in the case of more complex cell signaling pathway models, which may be described by hundreds or thousands of differential equations. An automated approach that could design experiments to test these complex systems has the potential to expand the scope of model selection experiments.

Previous work in designing dynamic stimuli for the purpose of model discrimination in systems biology has focused on choosing input trajectories that maximize the expected difference in the output trajectories of competing models [4–10]. In addition to model discrimination, a rich literature exists on experimental design in systems biology for the purpose of estimating model parameters [2,11–13]. These optimization approaches for model discrimination have been applied to small biological systems, but the nonlinearity of the models combined with the presence of many local minima has thus far limited their application [8].

**Figure 1:**
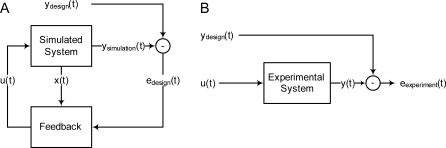
Schematic of Experimental Design (A) A feedback controller is used to solve for the stimulus *u*(*t*) that will drive the model system outputs *y*_simulation_(*t*) to follow the design trajectory *y*_design_(*t*). The inputs to the feedback controller are the deviation from the desired trajectory *e*_design_(*t*) as well as the model state *x*(*t*). (B) The designed stimulus can be applied to an unknown experimental system to assess the quality of the model. A stimulus based on a good model should be able to drive the experimental system output *y*(*t*) through the design trajectory.

There is a need to extend these methods to design experiments that may not be optimal but are capable of discriminating between large pathway models. Instead of trying to design an input signal that maximizes the predicted difference between two model readouts, we recast the problem as a control problem (Figure 1). We choose a target trajectory, and then challenge a model-based controller to drive the system to follow the target trajectory. The extent to which the controller based upon a given model is able to drive the physical system is a measure of the fitness of that model.

We demonstrate our methodology by applying it to the epidermal growth factor receptor (EGFR) pathway. This pathway has been extensively studied and modeled [14–18]. EGFR and its family members (Erb2, Erb3, and Erb4) are known to mediate cell–cell interactions in organogenesis and adult tissues [19]. Overexpression of EGFR family members is a marker of certain types of cancer, including head, neck, breast, bladder, and kidney [20]. Because of their clinical importance, the EGFRs themselves, as well as various downstream proteins, are targets of therapeutic intervention [21,22]. Despite clinical interest in the EGFR pathway and over 40 y of intense study, there is still much about the pathway that is not known. For example, in three recent studies [23–25], a number of proteins that changed phosphorylation state in response to EGF stimulation were found that were not previously known to be part of the pathway; in addition, many of the known pathway proteins are not part of any computational model [26].

The ordinary differential equation model of Hornberg et al. is a widely used mechanistic model of EGFR signaling [16]. This model is a refinement of earlier models of the pathway [17,18,27]. It describes signal transduction initiated at the cell surface by EGF binding to EGFR, leading eventually to the dual phosphorylation of ERK as the most downstream outcome, which then participates in a negative feedback to the top of the pathway. The elementary molecular processes modeled include bimolecular association and dissociation, phosphorylation and de-phosphorylation, synthesis and degradation, as well as endocytosis and trafficking all described with mass-action kinetics. The model contains 103 chemical species, 148 reactions, 97 independent reaction rates, and 103 initial conditions.

We applied our computational methods initially to a small portion of the EGFR model for development and demonstration purposes, and then to the full model. In both cases, we formulated a set of closely related models that exhibit similar step-response behavior. We built a controller capable of controlling each candidate model and asked the controller to drive the system output (doubly phosphorylated ERK) to a predetermined value. Finally, by applying these designed inputs based on the reference and perturbed models, we showed that it is possible to discriminate between the various model alternatives.

## Methods

### Model Formulation

In this work, we consider mass-action kinetic models consisting of zeroth-, first-, and second-order reactions described by ordinary differential equations. In the equations below, *k* signifies a rate constant; *A*, *B*, and *C* represent species or concentrations of species, depending on the context; and ∅ is the empty set or nothing.

Zeroth-order reaction:

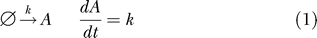



First-order reaction:

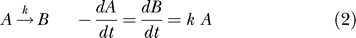



Second-order reaction:

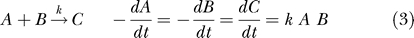



Large systems of reactions of this form can be represented compactly using Equation 4.

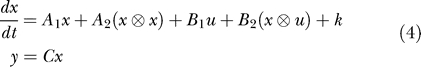
The state vector *x* describes the chemical species concentrations that are free to evolve in time according to the kinetics of the system. The input vector *u* represents the chemical species concentrations controlled by the experimenter. Matrices *A*_1_ and *B*_1_ represent first-order reactions, matrices *A*_2_ and *B*_2_ represent second-order reactions, and *k* represents constitutive (zeroth-order) reactions. The symbol ⊗ denotes the Kronecker product (also known as the matrix direct product) [28]. For vectors, this operator generates a vector of all quadratic products.

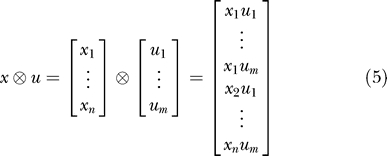



The output of the model *y* is a linear combination of the state variables represented by the matrix *C*.

### Control Formulation

A controller was developed to solve for the input signal *u*(*t*) that best achieves a particular objective in the output. We formulate this objective as a cost function *G*(*u*) that measures the distance between the model output and the desired output.

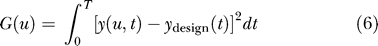



Here, *G*(*u*) is the sum of squares error between *y*(*u*,*t*), the model output for a given input *u*(*t*), and *y*_design_(*t*), the target output the controller is trying to match. *T* is the length of time of the experiment. The control problem is then to find an input function *u*(*t*) that minimizes *G*(*u*).

Equation 6 depends on models of the form of Equation 4, which are nonlinear and potentially high order. This prevents us from solving the minimization problem directly. To address this issue, we implement two different approximations. The first is based on controlling a model formed from successive linearizations of Equation 4 (henceforth referred to as the tangent linear controller), and the second is based on a local search of the input space (henceforth referred to as the dynamic optimization controller) [29].

### Tangent Linear Controller

A first-order approximation to Equation 4 at time *t* was computed by taking the Taylor series expansion about the current value of the state and input vectors (*x_t_* and *u_t_*).

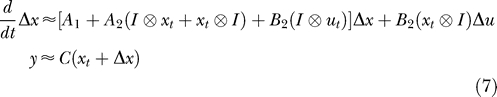



Equation 7 is a linear differential equation with state variable Δ*x* and time varying forcing term Δ*u*, which has both numerical and analytical solutions. However, this approximation would tend to diverge from the solution to Equation 4 with increasing Δ*t*, the time beyond the linearization point *t*, and (Δ*x*, Δ*u*), the distance from the linearization point (*x_t_*,*u_t_*). To mitigate this problem the true system (Equation 4) was propagated, and successive linearizations were applied to improve the controller performance. Effectively, the linearization point is allowed to slide along with the exact simulation.

Operationally, each time step was solved in three stages. First, the current state of the nonlinear simulation was used to derive a linear approximation about the current time point. Second, the linear system was solved to get the best input Δ*u*. The linear system was solved numerically by discretizing the input as a series of scaled and shifted boxcar functions [30] of width *τ*. Numerical integration with the MATLAB routine ode15s [31] was used to compute the system response to a unit boxcar input. The output of a linear time invariant system can be expressed as a linear combination of scaled and shifted impulse response functions. Thus, solving for the input was achieved by computing the weights to apply to the input pulses that gave the optimal output. This was solved as a linear system of equations with box constraints on the input to limit the maximum and minimum concentration using the MATLAB routine lsqlin. Third, the computed input signal was applied to the full nonlinear system for a short time step *τ*. The process was then repeated for the next time interval. Effectively, each step the algorithm solves for an input signal Δ*u* that is piecewise constant. The width of the intervals *τ* as well as the number of intervals is a parameter of the optimization and should be chosen based on the accuracy of the linear system.

### Dynamic Optimization Controller

In this controller formulation, rather than exactly solving the tangent linear system, we solved the full nonlinear problem iteratively using a gradient optimization method. Application of this method requires computation of the sensitivities of the least squares objective function (Equation 6) with respect to the input parameterization *p*. An efficient way to compute this quantity is to first solve for the adjoint sensitivities *λ* [29]. For the dynamical system (Equation 4) and the objective function (Equation 6), the adjoint equations are given by Equation 8.

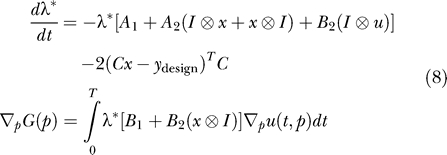



Here, λ* indicates the conjugate transpose. We use piecewise linear input functions described by parameters *p_i_*, which are the input function value at *T_i_*_,_ *u(T_i_)*; *u(t)* is then linearly interpolated between the control points at *T_i_*. For these piecewise linear input signals the *i^th^* component of the gradient 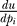 is given by:

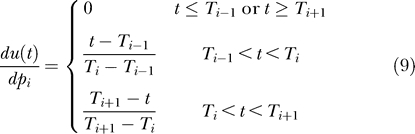



The adjoint equations were solved in MATLAB using ode15s [31] and the optimization was implemented using fmincon configured to use Quasi-Newton [32] with BFGS [33,34] in the MATLAB Optimization Toolbox Version 3.1.1.

### Constraining Input Signals

Thus far the input signals have been unconstrained, except by the choice of the discretization. However, in practice it may be desirable to restrict the space of input signals to those that could be feasibly achieved by a given experimental setup. For example, in many experimental setups it is easy to add material but difficult to take material away. Likewise, there may be a maximum and minimum concentration for the input signals, or a maximum rate of change for the input signal. We implemented these experimental constraints as linear inequality constraints of the form of Equation 10.

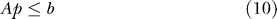
The matrix *A* and the vector *b* are passed as arguments to lsqlin in the case of the tangent linear controller, or to fmincon in the case of the dynamic optimization controller. An example of a linear constraint that might be applied is that the input increase monotonically. In this case, *A* and *b* are given by Equation 11.

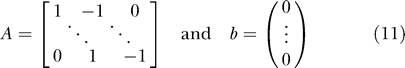



### EGFR Signaling Model

We based our model of EGFR signaling on that of Hornberg et al. [16], which itself is a refinement of earlier work [17,18,27]. The model contains 103 chemical species, and 148 elementary reactions; these reactions are of the type given by Equations 1, 2, and 3 and may be reversible. The model is parameterized by 97 distinct reaction rate values and 103 initial conditions. The details of this model are given in Dataset S3.

Here we also introduced a modified model of EGFR signaling, which contained six additional production/degradation reactions of the form of Equation 12, where *X* is one of (GAP, GRB2, SOS, RAS-GDP, SHC, or GRB2-SOS}.

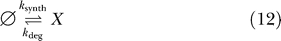



The degradation rate *k*_deg_ was set such that the steady-state value of the species was the same as the steady-state value in the unmodified model computed using Equation 13.

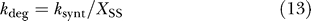



In addition to the protein synthesis and degradation reactions, a GAP-catalyzed turnover of RAS-GTP was implemented.

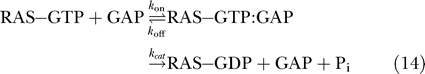



The rate constants (*k*_on_, *k*_off_, and *k*_cat_) are 5 × 10^−7^ cell molecules^−1^ s^−1^, 0.4 s^−1^, and 0.023 s^−1^, respectively. The rate constants *k*_on_ and *k*_off_ are taken from the analogous reaction where GAP is part of the receptor complex and the *k*_cat_ was fit so that the half–life of RAS-GTP in the absence of EGF matched literature values [35].

 Finally, a first-order turnover of internalized SOS was implemented with a rate constant of 10^−7^ s^−1^ based on the turnover rate of EGFR.

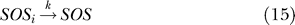



This augmented model has the additional property that if the input is removed (set to zero) it will return to its initial condition.

### MAPK Signaling Model

The mitogen activated protein kinase cascade is a signaling motif found repeated throughout biology [27]. In each step of the cascade a substrate is multiply phosphorylated by a kinase, which in turn is the input to the next layer in the cascade. The off signal, present in each layer, is a phosphatase that removes the phosphate groups. Despite knowing all of the species involved, the detailed mechanism of the enzymatic steps had been difficult to determine [27]. In particular, it was unclear if the kinase acted in two distinct enzymatic steps, whereby it released the substrate between phosphorylation steps (distributive mechanism) or if it performed both phosphorylation steps before releasing the substrate (processive mechanism).

A MAP kinase cascade consisting or RAF, MEK, and ERK is contained in the Hornberg EGFR pathway model. We extracted a tier of this cascade consisting of a single kinase, phosphatase, and substrate. The four reversible bimolecular reactions representing the phosphorylation of ERK by doubly phosphorylated MEK (MEKpp) and the de-phosphorylation by a phosphatase were used as the basis of a new model. The model contains a distributive dual phosphorylation step catalyzed by MEKpp and a distributive dual de-phosphorylation step catalyzed by a phosphatase. MEKpp is the system input; doubly phosphorylated ERK (ERKpp) is the output.

In addition to this basic model, three alternative models were constructed that differed in their mechanism of phosphorylation and de-phosphorylation (processive or distributive). The set of four models (distributive-kinase/distributive-phosphatase, processive-kinase/distributive-phosphatase, distributive-kinase/processive-phosphatase, processive-kinase/processive-phosphatase) represents all possible combinations of processive and distributive phosphorylation and de-phosphorylation mechanisms. The alternative models, which contain some rate parameters not included in the distributive-kinase/distributive-phosphatase base model, were parameterized by fitting the parameters to the step response of the double distributive model, which included both a step-up and a step-down experiment. The details of these four models are given in Dataset S2.

## Results

We have developed a method for designing an input signal capable of controlling the output of a candidate model. In practice, these input signals are useful for distinguishing among sets of candidate models.

### Simple Antibody-Binding Models

The dynamic optimization controller was applied to design input stimuli for each of the two alternative antibody binding reactions studied by Smith-Gill and co-workers [3]. For both the one-step and the two-step model (Dataset S1), the objective applied was to produce a constant output of antibody–ligand complex from time zero onwards. In the experiment performed by Smith-Gill and co-workers the measurement was a change in mass due to ligand binding as measured by surface plasmon resonance. While the fully bound complex is more stable than the postulated encounter complex, both have the same mass and would produce the same output signal. Therefore, in the case of the two-step model, the output is the sum of the encounter and fully bound complexes, whereas in the one-step model it is simply the fully bound complex. The basis set for the input was a 50-point piecewise-linear function with linear spacing. In the two-step model points were distributed evenly over the entire interval. In the one-step model points were placed evenly from 500 s to 600 s to accommodate the sharp transition.

**Figure 2:**
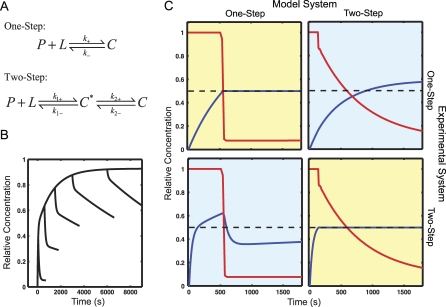
Analysis of Monovalent Antibody Binding (A) Two models of monovalent antibody binding, a one-step version with no intermediate, and a two-step version with an association intermediate *C* ^*^. (B) The results of six simulated experiments are shown as designed in [3]. Each trace is the response of the system to a square pulse of ligand concentration. The width of the pulse varies from 400 s to 6,000 s. The pronounced elbow in the middle curves is indicative of the two-state model. The one-step model cannot have compound off kinetics. (C) The set of experiments designed by this algorithm as well as simulated results are shown. Each pulse was designed to produce a level output when applied to the correct model (yellow boxes), which was observed, and produced a distinctly different result when applied to the other model (blue boxes). The red lines are the inputs (unbound *L*) the blue lines are the output (*C* or *C* + *C* ^*^). The smaller the gap between the blue and the black dashed line the better the model fits the real system. Looking across one row shows a pair of experiments that would be run together.

The results are shown in Figure 2. Both controllers designed an input signal that starts at high concentration to form complex quickly and then drops to a lower concentration to keep the complex from overshooting the desired value. However, the controller for the one-step model drops abruptly while the controller for the two-step model drops more gradually. The desired outputs were not recovered when the stimuli from the wrong models were applied. When the one-step input was applied to the two-step system, the output produced an undershoot followed by an overshoot. When the input designed for the two-step model was applied to the one-step system, the complex concentration also produced an overshoot, but one that persisted. In both cases, accounting for the presence or absence of the encounter complex was critical for controlling the output correctly.

It is interesting to note that this method allows for the selection of both the more complex model (if it is correct) as well as the simpler model. This is not possible using standard a posteriori metrics, such as least squares, which will always favor the more complex model. While there are methods that try to correct for this bias [36], properly accounting for model complexity in large nonlinear systems remains an open problem [37]. Comparing our results to the Smith-Gill pulse method (Figure 2B), it is clear that both computational experiments permit the two models to be distinguished in favor of the two-step method. However, for larger and more complex cases, it is unclear whether intuitive approaches or square pulse inputs will be sufficient to design distinguishing experiments. Another feature of the simulations is that the designed pulse produces a level output that does not require fine time resolution to accurately measure. This can be a significant advantage for more complex experimental systems, such as cell signaling measurements, where limitations on experimental observations are even more severe, whether in terms of numbers of species, time points or other factors.

### MAPK Signaling

Mitogen-activated protein kinase cascades have been extensively studied experimentally and modeled computationally. While many variants exist, the canonical pathway consists of three layers of kinases and phosphatases. For each layer, the kinase activates the downstream kinase by dual-phosphorylation and the phosphatase deactivates the downstream kinase by removing the phosphate groups. Knowing the general structure of this pathway, it was still difficult to determine the details of the enzymatic steps. In particular, it was unknown if the kinase acted in a processive mechanism (adding both phosphate groups in a single step), or if it acted in a distributive mechanism (adding the phosphates in two distinct enzymatic steps). The difficulty arose from the fact that, without measuring all of the phosphorylation forms, both mechanisms could fit the step response data. The issue was eventually resolved by devising an experiment that could separate all of the phosphorylation forms [27]. Here we show that, in principle, the mechanisms could have been distinguished using our method, without adding additional measurements.

**Figure 3:**
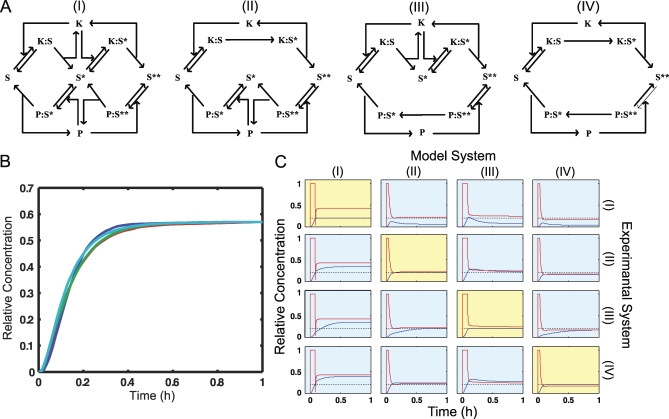
Analysis of MAPK Mechanisms (A) Four alternative MAPK reaction schemes are shown. These correspond to all combinations of processive and distributive kinase and phosphatase mechanisms. Model I is the canonical all-distributive mechanism. For each model the input is the concentration of activated kinase (*K*) and the output is the doubly phosphorylated substrate (S**). (B) All four MAPK models respond in very similar fashion to a step increase in kinase input (*u* = 1). (C) A set of 16 model-selection experiments. Each row is a different experimental system and each column is a different candidate model. Red lines are inputs (activated *K*), blue lines are outputs (S**), and the black dashed line is the design output trajectory. The experiments on the diagonal show that the correct model can control the system. The off-diagonal experiments show that the wrong model does a worse job. This difference can be used to select the correct model.

To address this problem we generated four candidate models of a MAPK dual phosphorylation reaction. All four models contained forward phosphorylation and reverse de-phosphorylation steps, but differed in the detailed mechanisms. For both the forward and the reverse reactions we considered a processive (one-step) and a distributive (two-step) mechanism (Figure 3A). Taking all combinations of distributive and processive reactions produced four models. For each model the free kinase concentration was the input variable and the concentration of doubly phosphorylated substrate was the output.

For each of the four models, a stimulus was developed using the tangent linear controller. The objective was to drive the output to a fixed value that remained constant with time. Each of the four designed signals was used to stimulate each of the four models, and the resulting 16 experiments are shown in Figure 3C. Along the diagonal, one can see that the input signal derived from the correct model was able to effectively control the system. However, looking at each off-diagonal entry shows that inputs from each wrong model did a poor job controlling each system. In any real experiment, there is only one true system, which corresponds to performing the experiments from a single row of the figure.

As with the antibody models, the algorithm was able to find a set of signals that distinguished amongst multiple models. It is worth noting that these solutions were generated automatically from the candidate models and did not require explicit user supervision.

### EGFR Pathway

**Figure 4:**
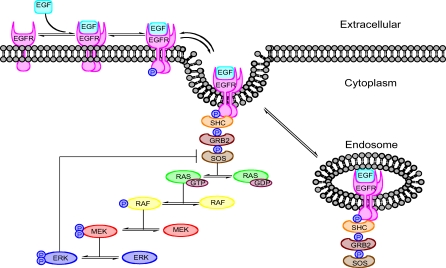
Schematic of EGF-Induced Signaling This schematic shows the major steps in EGFR signaling. At the top of the pathway ligand binds to the receptor and induces receptor dimerization and activation. The signal is then transduced through a series of adaptor proteins SHC, GRB2, and SOS, which in turn activates the MAPK cascade RAF, MEK and ERK. There are two negative feedback loops: internalization and degradation of the receptor complex, and ERK deactivation of SOS.

A popular ordinary differential equation model of the EGFR pathway is that of Hornberg and co-workers [16]. This model consists of 103 differential equations and includes ligand binding, receptor dimerization and activation, adaptor protein binding, trafficking of the receptor complex, and activation of the MAPK cascade terminating with ERK dual phosphorylation (Figure 4). This model was built as a set of successive refinements of earlier models [17,18,27], with each refinement adding a new level of detail to the model. In its most recent formulation an additional negative feedback loop was added whereby activated ERK phosphorylates SOS and deactivates it. This model has been shown to agree with time course data collected in cell based assays as well as literature values for parameters measured in vitro [18]. We compare the original Hornberg model to a version with additional changes. We continue this model evolution by modifying the Hornberg model so that, when the input (EGF) is removed, the model returns to its initial conditions. This reset behavior is observed experimentally. Cells cultured in media containing EGF but switched to serum and EGF free media 12 h before stimulation, are able to respond to a dose of EGF added to the media [23]. This indicates that after EGF has been removed, the pathway returns to an EGF responsive state.

In the Hornberg model, the dominant mechanism for desensitization and adaptation of the pathway to EGF is endocytosis and degradation of the receptor complex. Opposing this process are constitutive production and degradation reactions for the receptor, which allow the receptor level to return back to steady state after stimulation. This same process degrades other proteins in the receptor complex GAP, GRB2, SOS, and RAS, but the current model does not contain synthesis terms for these proteins. As a result, prolonged stimulation depletes these proteins and prevents the activation of RAF, MEK, and ERK. We added production and degradation reactions analogous to the reactions for the receptor for all of the proteins in the receptor complex. Rate constants were chosen such that the steady-state levels in the absence of stimulation were the same as the initial conditions for the model and the exponential time constant for the approach to steady state was the same as for EGFR.

The second modification to the model was in the RAS-GDP/RAS-GTP cycle. In the Hornberg model, activated receptor is needed to catalyze the recycling of RAS-GTP* (a molecule of RAS-GTP that has already activated a molecule of RAF) that is waiting to be recycled to RAS-GDP. If EGF is removed, RAS can be trapped in the RAS-GTP* form, preventing the system from returning to steady state. We addressed this by adding an additional enzymatic step to recycle RAS-GTP* back to RAS-GDP catalyzed by GAP and parameterized using literature rate constants [35].

**Figure 5:**
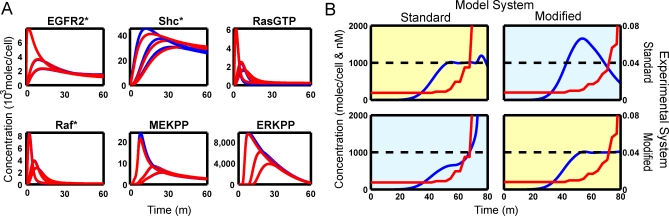
Comparison of Step Experiment to Designed Experiment for EGFR Pathway with Original and Modified Models (A) Both models respond similarly but not identically to a step input in EGF over a range of concentrations. The red lines are the outputs of the modified model and the blue lines are the outputs of the standard model. Often the blue lines are not visible because they are under the red lines. (B) Two designed dynamic stimuli were applied to two models of the EGFR pathway. The red lines show the input (EGF) concentration as a function of time. The blue lines show the output concentrations (ERKpp). The dashed black line shows the target ERKpp concentration. The controller for the standard model is unable to keep the output level high and saturates. In contrast, the modified model requires a more gradual increase in the input and can control the experiment over the entire time course. In both cases the controller based on the wrong model performs worse that the controller based on the correct model.

With the addition of these new reactions, the modified model returns to its initial conditions after stimulation. For the remaining model parameters (the parameters shared with the original model) we fit the modified model to the original using data from a simulated step-response experiment (Figure 5A) constraining them to be within 10% of their original value. Despite the introduction of these new mechanisms and the tight constraints on the parameters, the step responses of the six molecular species modeling those presented in the original paper [18] (Figure 5B) are very similar in the original model (blue curves) and the modified model (red curves). The largest difference is in the SHC* time course, which has a very similar shape and varies by at most 11%. While significant, this difference would be very difficult to detect in a standard biological experiment. As such, the modified model is a reasonable alternative to the original model, and it would be hard to reject either mechanism using the step-response data alone.

From this starting point we used our methodology to design an experiment that could distinguish between the current model and the modified model of the EGFR pathway. For each model we tasked the dynamic optimization controller with driving the concentration of doubly phosphorylated ERK to a constant level of 10^4^ molecules per cell. The input basis set was 25 points linearly spaced over the interval. To model the experimental condition where it is easy to add EGF to the dish of cells but difficult to remove, we implemented a monotonicity constraint. Figure 5B shows the inputs designed for each of the two models applied to each system with the resulting ERKpp time courses. Due to the negative feedback loops, both models required a steadily increasing concentration of EGF to maintain a constant level of ERKpp. However, the original model was much more difficult to control; as time progressed increasingly high doses of EGFR were required to maintain a constant output. The modified model required a much gentler increase in EGF concentration to maintain its level and was able to keep the concentration of ERKpp high to the end of the time period. Trial calculations showed that this result was robust to order of magnitude changes in the new rate parameters introduced in the modified model. Applying these two signals in an experiment could be used to distinguish between these two models, as demonstrated by the simulations.

## Discussion

The most common stimulus-response protocol involves applying a step change in one or more input concentrations and following the evolution of one or more downstream molecules. For a linear system, this type of experiment can provide enough information to fully identify the system [38]. However, even simple biochemical systems are nonlinear, and as such there is no a priori reason to believe that a step-response experiment will be sufficient to uncover the relevant dynamics of the system and allow for the selection of a unique model. As a result, it is often possible, if not probable, that multiple mechanisms fit the same set of step-response data. We have shown here that using dynamic stimulation can improve stimulus-response experiments. Even in the context of complex pathways with limited numbers of inputs and outputs, experiments can be designed that are capable of distinguishing amongst alternative mechanisms. Moreover, for the EGFR pathway studied, the differences detected were in the middle of the pathway, far from the location of the stimulus or the readouts.

One possible explanation for the results presented here is that we have stimulated the systems with high-frequency signals, and it is this fact that allows for model discrimination. While the high-frequency content almost certainly plays a part, the fact that differences between models are observed at low frequency distinguishes our results from other standard test signals. For example, in linear systems it is common to use random or pseudorandom signals to discriminate among models. Figure S1 shows such an experiment. While the signal is discriminating, the observed differences are high frequency and would be difficult to distinguish in a standard biological assay, which is usually sampled sparsely in time.

Formulating experimental design as a control problem yielded a relatively straightforward numerical solution, which allowed us to apply our method to large pathway models. While the method does not yield optimal experiments, in the sense of maximizing the least squares error between model ouputs, the results are still of practical benefit and appear sufficient to distinguish amongst model candidates. In the systems studied here, the designed inputs were able to substantially increase the differences observed between competing models when compared to the corresponding step-response experiment. By prescribing the target output trajectory, it should be possible to tailor the experiments to the available measurement methods, thereby achieving the most benefit from existing assays. It is worth noting that in all of the examples presented here, the target function was a constant output concentration. This was chosen for simplicity rather than for any special property of these targets. The problem of the best target function is an interesting one but is beyond the scope of this work. However, in Figure S2 we show calculations for the antibody–ligand system using other simple target functions, lines of constant slope, and find that designed inputs based on these signals have similar discriminating power.

In each of the cases presented here, the dynamic stimuli allowed us to select the correct mechanism from a set of plausible candidates. However, it is possible that for a particular system and set of constraints, the algorithms presented here may fail to find a signal that is sufficiently discriminating. In this case a different choice of target function or a more sophisticated optimization approach may yield better results. However, it is worth noting that in the systems studied here both methods were able to find very good solutions in all cases. In general, the tangent linear controller was more computationally efficient and yielded smoother signals, whereas the dynamic optimization controller was slower but did not require tuning of parameters such as *τ*.

One potential limitation of our method comes from our reliance on parameterized models. The accuracy of the parameterizations will affect the quality of the predictions made by the controllers and thus the ability to distinguish between models. To demonstrate this, we generated 100 different parameterizations of the one-step and two-step antibody models and then applied the control signals designed using the nominal parameter set (Figure S3). The parameter variation resulted in output trajectories that were quantitatively different from the predicted output trajectories. However, the overall shape of the output trajectories was preserved.

All of the results presented here were in simulation. In practice, experimental error and measurement noise will make it more difficult to distinguish between models. As a result, one may only be able to effectively discard some candidate models, and reduce the pool of hypotheses. However, these experimental challenges also motivate our method, as it has the potential to increase the experimental observability of model differences when compared to a more traditional experiment, such as a step response. Moreover, the fact that potential mechanisms can be evaluated without having to resort to additional inputs or outputs is especially valuable in laboratory experiments, where adding additional inputs or outputs may require significant effort, such as developing new experimental reagents.

## Supporting Information

Dataset S1 Models of the One-Step and Two-Step Antibody–Ligand Binding Reaction (10 KB TAR)

Dataset S2 Models of the MAPK Phosphorylation and De-Phosphorylation (10 KB TAR)

Dataset S3 Models of the Epidermal Growth Factor Signaling Pathway (50 KB TAR)

Figure S1 Random-Pulse Experiment This figure shows a series of pulses as input to the one-step and two-step antibody–ligand models for two different distributions of switching time. In the slower switching time (A) the input signal changes at random with a mean of 900 s, and in the faster switching time (C) the input switches at random with a mean of 90 s.
(B) The response of the two models to the slower input shows that the one-step model looks like a smoothed out version of the two-step model.
(D) The trend is similar with the faster varying signal.
(883 KB EPS)

Figure S2 Matching Different Target Functions The dynamic optimization controller was used to design input signals to drive the one-step and two-step antibody–ligand models. The target functions (black dashes) are a set of lines of increasing slope. The input is the concentration of free ligand (red) and the output is the amount of complex formed (blue). The figures on the diagonal show that the controller based on the correct model is able to accurately drive the systems to follow the target functions very closely. Whereas, the figures on the off diagonal show that the inputs based on the wrong models cause either large over- or undershoots of the target function.
(1.2 MB EPS)

Figure S3 The Effect of Parameterization Errors A dynamic optimization controller was constructed based on a nominal parameterization of the one-step and two-step antibody models. The resulting input signal is shown in red and the output is shown in dark blue. These input stimuli were then applied to a set of 100 different parameterizations of these two models (cyan lines). The parameters were chosen from a log normal distribution with a mean centered on the nominal value and a variance of 10%.
(10.8 MB EPS)

## Abbreviations

EGF: epidermal growth factor
EGFR: epidermal growth factor receptor
ERK: extracellular signal–related kinase
ERKpp: doubly phosphorylated ERK
GAP: GTPase activating protein
GRB2: growth factor receptor bound protein 2
MAPK: mitogen-activated protein kinase
MEK: MAP kinase/ERK kinase
MEKpp: doubly phosphorylated MEK
ODE: ordinary differential equation
SOS: son of sevenless
